# Dietary pattern, inflammation and cognitive decline: The Whitehall II prospective cohort study

**DOI:** 10.1016/j.clnu.2016.01.013

**Published:** 2017-04

**Authors:** Mio Ozawa, Martin Shipley, Mika Kivimaki, Archana Singh-Manoux, Eric J. Brunner

**Affiliations:** a Department of Epidemiology and Public Health, University College London, London, UK; b INSERM, U1018, Centre for Research in Epidemiology and Population Health, Villejuif, France

**Keywords:** Diet, Inflammation, Cognitive decline, Aging, Longitudinal study, IDP, inflammatory diet pattern

## Abstract

**Background & aims:**

Low-grade inflammation appears to play an etiological role in cognitive decline. However the association between an inflammatory dietary pattern and cognitive decline has not been investigated. We aimed to investigate dietary patterns associated with inflammation and whether such diet is associated with cognitive decline.

**Methods:**

We analyzed 5083 participants (28.7% women) from the Whitehall II cohort study. Diet and serum interleukin-6 (IL-6) were assessed in 1991–1993 and 1997–1999. We used reduced rank regression methods to determine a dietary pattern associated with elevated IL-6. Cognitive tests were performed in 1997–1999 and repeated in 2002–2004 and 2007–2009. The association between dietary pattern and cognitive decline between ages 45 and 79 was assessed using linear mixed models.

**Results:**

We identified an inflammatory dietary pattern characterized by higher intake of red meat, processed meat, peas and legumes, and fried food, and lower intake of whole grains which correlated with elevated IL-6 both in 1991–1993 and 1997–1999. A greater decline in reasoning was seen in participants in the highest tertile of adherence to the inflammatory dietary pattern (−0.37 SD; 95% confidence interval [CI] −0.40, −0.34) compared to those in the lowest tertile (−0.31; 95% CI −0.34, −0.28) after adjustment for age, sex, ethnicity, occupational status, education, and total energy intake (p for interaction across tertiles = 0.01). This association remained significant after multivariable adjustment. Similarly for global cognition, the inflammatory dietary pattern was associated with faster cognitive decline after multivariable adjustment (p for interaction across tertiles = 0.04). Associations were stronger in younger participants (<56 years), reducing the possibility of reverse causation.

**Conclusions:**

Our study found that a dietary pattern characterized as higher intake of red and processed meat, peas, legumes and fried food, and lower intake of whole grains was associated with higher inflammatory markers and accelerated cognitive decline at older ages. This supports the case for further research.

## Highlights

• The derived inflammatory dietary pattern was associated with repeated signs of inflammation.
• The derived dietary pattern is characterized by higher intake of red meat, processed meat, peas and legumes, and fried food, and lower intake of whole grains.
• This derived dietary pattern associated with systemic inflammation.
• This inflammatory dietary pattern predicted faster decline in reasoning over ten years of follow-up.

## 1 Introduction

The number of people with dementia worldwide was estimated at 35.6 million in 2010. It is predicted to reach 65.7 million in 2030, and 115.4 million in 2050 [1]. When symptoms of neurodegeneration appear, changes in the brain have already started and it may be too late to take steps to prevent or delay disease onset. Therefore, it is important to identify potential early interventions. Success in this objective would reduce dementia prevalence and increase the proportion of older people able to live independently in the community.

Inflammation has been recognized as a risk factor for age-related neurodegenerative diseases including cognitive impairment, Alzheimer's disease, and vascular dementia [2], [3]. In epidemiological studies higher levels of circulating inflammatory markers, especially interleukin-6 (IL-6) are associated with greater cognitive decline [4], [5]. Diet influences the function of immune systems. High consumption of vegetables and fruit or a diet rich in antioxidants, for example, has been shown to reduce systemic inflammation [6]. In contrast, dietary patterns characterized by higher intakes of red and processed meats, sweets, desserts, french fries, and refined grains, appear to increase inflammation [7]. The role of diet as a determinant of age-related cognitive decline and dementia however remains uncertain.

A recent randomized controlled trial showed improved or maintained cognitive function in the intervention group (diet, exercise, cognitive training and vascular risk monitoring), compared to the control group [8]. This finding is encouraging but because the trial involved a multi-factorial intervention, the contribution of the dietary component is indeterminate. Observational studies suggest that a Mediterranean-type dietary pattern confers advantage but, as in the trial, the active ingredients and mechanisms responsible for cognitive protection across the spectrum of severity remain unknown [9].

In this study, our aim was to examine the associations of diet pattern with inflammatory markers and changes in cognitive function within a single analytic setting. We hypothesized that a dietary pattern related to increased IL-6 concentration in midlife predicts accelerated cognitive decline in a large sample of middle-aged population.

## 2 Materials and methods

### 2.1 Study populations

The Whitehall II cohort is an ongoing study established in 1985 among 10,308 (67% men) British civil servants. The detail of the cohort and follow-up has been described previously [10]. Briefly, all participants aged 35–55 years in 20 Civil Service departments in London, UK, were invited to participate in this study. Of those invited, 73% participants agreed to participate in the baseline survey which involved a clinical examination and self-administered questionnaire. Subsequent follow-up clinical examinations have taken place in 1991–1993 (phase 3), 1997–1999 (phase 5), 2002–2004 (phase 7), and 2007–2009 (phase 9).

### 2.2 Cognitive function measurement

3 clinical examinations over 10 years were administered for the cognitive test battery (in 1997–1999, 2002–2004, and 2007–2009), and consisted of 4 standard tasks: Alice Heim 4-I, short-term verbal memory, phonemic fluency, and semantic fluency [11]. The Alice Heim 4-I is consisted of a series of 65 verbal and mathematical reasoning items of increasing difficulty [12]. It tests inductive reasoning, measuring the ability to identify patterns, and to infer principles and rules. 10 min were given to do this section. Short term verbal memory was assessed with a 20 word free recall test. Participants listened to a list of 20 single- or double-syllable words at 2 s intervals and then had to recall and write them down in 2 min. We used two measures of verbal fluency: phonemic and semantic fluency [13]. Participants were asked to recall in writing as many ‘S’ words and as many animal names as they could in 1 min. Global cognitive score was created to provide a summary score of these 3 tests by taking a mean of the z scores of each test. This method has been shown to minimize problems due to measurement error on the individual tests [14]. The Mini-Mental State Examination (MMSE) was administered in 2002–2004 and 2007–2009. MMSE is a measure of global cognitive function with a 30-point range.

### 2.3 Nutritional survey

Dietary intake was assessed by using a 127-item food frequency questionnaire. The validation of this questionnaire with 7-day diary has been reported previously [15]. For each of the food items, participants self-reported how often on average they had consumed a common unit or portion size in 1 of 9 categories during the previous year. To reduce misclassification, we used the data collected in 1991–1993 and 1997–1999, and calculated the average intake of each food and nutrient. Each food group was adjusted for energy intake using the density method.

### 2.4 Inflammatory markers

In 1991–1993 and 1997–1999, fasting serum was collected between 8 AM and 1 PM and stored at −80 °C. IL-6 was measured with a high-sensitivity ELISA (R&D Systems, Oxford, UK). Values lower than the detection limit (0.08 pg/mL) were set to half the detection limit.

### 2.5 Covariates

Demographic variables included age, sex, ethnicity, education, and total energy intake. Health related variables included body mass index (kg/m2), diabetes mellitus, hypertension, smoking, and leisure time physical activity. Ethnicity (white, south-Asian, black, other), occupational position (6 levels), education (less than primary school, lower secondary school, higher secondary school, university, and higher university degree), smoking history (never, ex-smoker, or current), and leisure time physical activity (physically active corresponds to more than 2.5 h/wk of moderate physical activity or more than 1/wk of vigorous physical activity) were based on self-report. Body weight and height were measured, dressed in a cloth gown and underclothes, in standardized fashion and body mass index (kg/m2) was calculated. Diabetes mellitus was defined by fasting glucose ≥7.0 mmol/L or a 2 h post load glucose ≥11.1 mmol/L, self-reported physician-diagnosed diabetes, or use of diabetes medication. Hypertension was defined as systolic blood pressure ≥140 mmHg or diastolic blood pressure ≥90 mmHg, or use of antihypertensive drugs. Since alcohol consumption was included as one of the food components in our dietary pattern, we did not include alcohol in covariates.

### 2.6 Statistical analysis

The dietary pattern associated with cognitive decline was assessed using reduced rank regression [16]. This method identifies linear functions of food groups, i.e. the dietary pattern, to maximize explained variation in one or more intermediate response variables. We selected serum IL-6 measurements in 1991–1993 and 1997–1999 as response variables on the basis of the known direct association of circulating IL-6 level with cognitive decline in our study and others [4], [5]. The dietary pattern related to IL-6 level was derived based on 37 predefined food groups [17]. The scores for dietary pattern were categorized in tertiles.

A linear mixed model was used to estimate the association between dietary pattern and cognitive decline over 10 years [18]. This method uses all available data over the 10 years of follow up, including participants with an incomplete set of values for cognitive tests, and takes into account the correlation of the repeated measurements on the same individual. In our analyses, age in years was used as the time variable. We included intercept and slope as random effects to allow individuals to have a different cognitive function score at baseline and different rate of cognitive decline during follow-up. Analyses were adjusted for demographic variables (age, sex, ethnicity, education), total energy intake, and health related variables (body mass index, diabetes mellitus, hypertension, smoking, and leisure time physical activity), and interactions between each variable and time. The <variable> X time interaction term represents the effect of <variable> on cognitive change over time.

Logistic regression analysis was used to identify the association between derived dietary pattern and decline in MMSE, defined as reduction of 3 points or more in MMSE from 2002-2004 to 2007–2009. Decline of 3 or more MMSE points has been reported as a reliable change [19]. The trends in the mean values or the frequencies of risk factors across dietary tertiles were tested by using linear and logistic regression, respectively.

We conducted several sensitivity analyses. We excluded the participants diagnosed with diabetes mellitus and hypertension to exclude misclassification since participants with diabetes and hypertension might have changed their dietary habits at the baseline making diabetes mellitus and hypertension potential confounders. We also excluded participants with the lowest 10% score on global cognition to exclude potential cases of dementia, and to avoid reverse causation on dietary habits at baseline due to low cognitive function. In addition, we further conducted a stratified analysis with median age and the BMI to evaluate whether the association would be different across the strata. Two-sided p < 0.05 was considered statistically significant in all analyses. The software package SAS (version 9.3; SAS Institute, Cary, NC, USA) was used for all statistical analyses.

### 2.7 Ethics

Written informed consent was given by all participants, and the University College London ethics committee approved this study.

## 3 Results

**Fig. 1:**
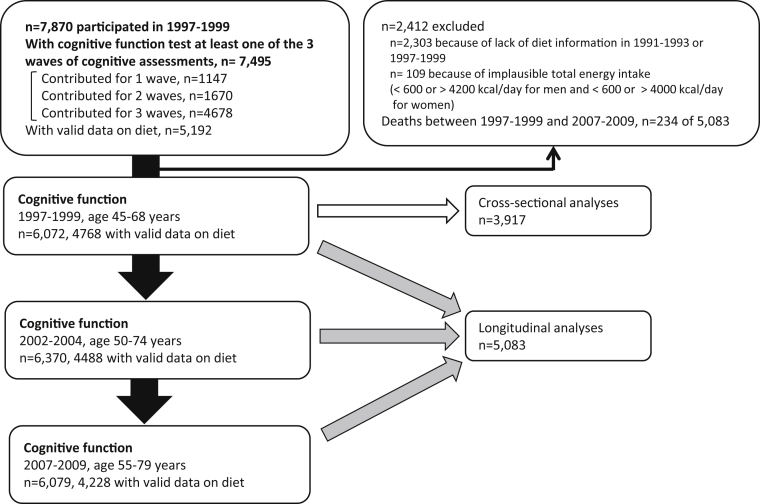
Study profile.

A total of 7870 participants completed the medical examination in 1997–1999, the baseline of this study. Of these, 7495 individuals participated in at least one of the three cognitive function assessments over the following 10 years. Of these, 4678 (62.4%) contributed to all 3 phases, 1670 (22.3%) to 2 phases, and 1147 (15.3%) to a single phase. Participants with missing diet assessment data in 1991–1993 and 1997–1999 (n = 2303), and participants with implausible total energy intake (<600 or > 4200 kcal/day for men and <600 or > 4000 kcal/day for women) (n = 109) were excluded, such that 5083 participants remained in the analysis (Fig. 1).

The mean age of the participants at the baseline was 56 years, and the proportion of women was 28.7%. The prevalence of diabetes and hypertension at baseline was 4.4% and 28.2%, respectively. Among the participants, 8.7% were currently smoking, and 53.0% reported moderate and/or vigorous physical activity during leisure time.

Two dietary patterns were identified using reduced rank regression. Scores for these two patterns explained 3.54% of the total variation of the response variables (IL-6 in 1991–1993 and 1997–1999). The first dietary pattern accounted for 95% of explained total variation. Thus, we named the first dietary pattern the inflammatory dietary pattern (IDP) and used this for further analyses. IDP score positively correlated with IL-6 both in 1991–1993 (r = 0.08, p < 0.001) and IL6 in 1997–1999 (r = 0.13, p < 0.001) adjusting for age and sex.

The factor loadings of the 37 food groups represent the magnitude and direction of each food group's contribution to IDP (Table 1). A positive factor loading value indicates that a higher IDP score is associated with an increased intake for that food group. The food groups with a factor loading of 0.25 or more were red meat, processed meat, peas and legumes, and fried food, while the food group with a factor loading of −0.25 or less was whole grains. The range of factor loading was −0.47 (for whole grains) to 0.27 (for red meat). The correlation coefficients between food groups and IL-6 in 1991–1993 and 1997–1999 are shown in Supplemental Table 1.

Table 2 shows characteristics of study participants according to the tertile of IDP score at baseline. Mean values of IL-6 increased according to the higher adherence to IDP in 1991–1993 and 1997–1999. Participants with higher IDP score were likely to be older, more likely to be women and to have hypertension, diabetes, and smoking history. The mean values of systolic and diastolic blood pressure and body mass index increased with higher IDP score. Energy intake was lower in participants with higher IDP score.

Table 3 shows mean cognitive decline with 95% confidence intervals (CI) over 10 years according to the tertiles of IDP score. In the model adjusted for age, sex, ethnicity, occupational position, education, and total energy intake, compared to the lowest tertile of IDP score (−0.31, 95% CI −0.34, −0.28), those in the highest (−0.37, 95% CI −0.40, −0.34; p = 0.005) tertiles experienced faster cognitive decline across the tertile in reasoning. This association remained significant after further adjustment for body mass index, diabetes mellitus, hypertension, smoking history, and leisure time physical activity. A similar pattern was seen between the highest and lowest tertiles of IDP for memory although the difference was not significant (p for interaction across tertiles = 0.40). Verbal fluency showed no association with IDP score. In global cognition, compared to the lowest tertile of IDP score (−0.31, 95% CI −0.33, −0.28), those in the highest (−0.35, 95% CI −0.38, −0.32) tertiles experienced faster cognitive decline in the multivariable adjusted model (p = 0.03).

In cross-sectional analyses (Table 4), IDP score at baseline and decline of 3 points or more in MMSE were significantly associated with age, sex, ethnicity, occupation, education, and total energy intake adjusted model. This association attenuated after further multivariable adjustment.

In sensitivity analyses, we excluded the participants diagnosed with diabetes mellitus and hypertension and this did not change the results (data not shown). Excluding participants with the bottom 10% score of global cognition also did not change the results (data not shown). Furthermore, in stratified analyses with median age (56 years), higher IDP score showed significantly faster cognitive decline in reasoning in the age<56 group, but the association was not significant in the ≥56 age group (Supplemental Table 2). In addition, we stratified the analyses using median body mass index. The association between IDP and cognitive decline was similar in both strata (Supplemental Table 3).

## 4 Discussion

In this large cohort of middle-aged men and women, a dietary pattern associated with systemic inflammation at two measurement occasions five years apart predicted faster decline in reasoning over ten years of follow-up. The inflammatory dietary pattern was associated with repeated signs of inflammation and is characterized by higher intake of red meat, processed meat, peas and legumes, and fried food, and lower intake of whole grains. This is, to the best of our knowledge, the first longitudinal study to link diet, inflammation and risk of cognitive decline over 10 years within a single analytic setting. This may support the mechanisms that an inflammatory diet pattern increases the risk of faster cognitive decline through low-grade systemic inflammation, and this gives caution and provides helpful information to the general public in choosing their daily food to eat.

So far, dietary pattern analyses have generally been conducted using either a principal components analysis that determines, post hoc, dietary patterns specific to the targeted population or a hypothesis-oriented approach based on an ideal dietary pattern e.g. the Mediterranean diet. Principal components analysis reflects the dietary background but is not able to include any evidence about predictors of targeted disease risk. On the other hand, hypothesis-oriented analyses of an a priori dietary pattern are based on previous information but ignore the dietary background of the targeted population. So it is beneficial to use reduced rank regression since the derived dietary patterns combine the advantage of both approaches. The approach incorporates evidence about predictors and reflects the dietary background of the targeted population.

Reduced rank regression has been employed with nutrients as hypothesized intermediate or response variables to identify a dietary pattern which can be tested as a predictor of dementia risk [20], [21]. One study from the U.S. and another from Japan used fatty acid fractions, and selected vitamins and minerals. Despite differing food cultures, the reduced rank regression technique succeeded in extracting a dietary pattern in each study which reflected the predictor information and which was associated with the risk of dementia. These studies showed that reduced rank regression is a powerful tool to test dietary hypotheses based on etiology [20], [21]. Our novel study also tested a dietary hypothesis but, rather than nutrients, used serum IL-6 as the predictor of the dietary pattern.

A previous study based on an a priori index of diet-related inflammation did not find an association with Alzheimer's disease or with serum C-reactive protein (CRP) [22]. In contrast, we derived an IDP empirically, based on correlations of food groups with an inflammatory marker, showing that the IDP predicted rate of cognitive decline (reasoning) in a second independent analytic step.

We chose IL-6 as the response variable, since our previous study suggested an association between IL-6 and cognitive function that was not evident with CRP [5]. Some reports, but not all, are consistent with our finding [4], [5]. Although it remains unclear which inflammatory markers associate most strongly with cognitive function, the association with IL-6 is consistently stronger than with CRP in neuroimaging studies [23].

The mechanisms underlying the proposed association between an inflammatory dietary pattern and cognitive function are likely to involve several processes. Although the IDP diet is not an immediate cause of cognitive decline, to the extent that dietary pattern may produce systematic inflammation, several lines of research suggest pathological pathways involving inflammatory processes may operate. Animal studies indicate that deficits in cognitive function with increased amyloid-β deposition are due to elevated oxidative stress and inflammation [24]. Moreover, systemic inflammation might influence cognitive function through thrombotic effects [25]. Higher adherence to the IDP increases inflammation, and higher inflammation may cause subsequent cognitive decline due to its neurotoxicity, which has a direct effect on the brain. Additionally, a high IDP may increase cardiovascular risk by inducing vascular and metabolic changes, as well as inflammatory abnormalities.

Depression, which is a risk factor for dementia [26], can act both as a mediator and a confounder of the relationship between diet and cognitive decline. In its mediating role, incident depression has been linked with an inflammatory dietary pattern [27] and, further, systemic inflammation is associated with higher risk for depression. In the role of potential confounder, participants with depressive symptoms may have higher risk for faster cognitive decline, and may modify their diet secondary to their low affect. Because of its likely mediating role, we did not control for depressive symptoms in the main analysis. However after additional adjustment for depression the effect of inflammatory diet on cognitive decline was only marginally attenuated (2.9%).

The derived IDP is consistent with previous studies showing associations between overall dietary pattern, food group intake and inflammatory markers [28]. Western-type diets have been linked to increased systemic inflammation [7]. Common components of the western dietary pattern and our IDP which was positively associated with inflammation include red meat and fried food. Further, the IDP and western diet are both characterized by low intakes of unrefined plant foods, including whole grains, and thus of antioxidant nutrients. Epidemiological and experimental studies provide reasonable evidence that this combination of pro-inflammatory dietary elements together with a relative lack of antioxidant intake may increase risk of dementia [7]. In our study, whole grain intake was strongly and inversely associated with the IDP, and there was a high negative correlation with IL-6 levels at both measurement occasions. Therefore, lower intake of whole grain which contains greater antioxidants may induce our IDP to be associated with faster cognitive decline. Fruit and vegetables are also great sources of antioxidants. However in our current study, these food groups were not associated with our derived dietary pattern IDP.

Paradoxically, we found that intake of peas and legumes loaded strongly on the IDP factor. This finding conflicts with expectations for a dementia-protective diet [29]. We considered that participants may have been describing consumption of baked beans as a major component of legume intake, in combination with pro-inflammatory foods. This association was confirmed, the intake of peas and legumes as a food group is highly correlated with intake of baked beans (correlation coefficient = 0.60, p < 0.0001). Baked beans are often consumed as part of an English-style breakfast with processed meats including bacon and other fried foods. The strongest correlations between baked beans and other food groups were 0.16 with fried food, and other correlations for each food groups were processed meat 0.10, fried food 0.16, sugar beverages 0.10, and condiments 0.13 (food groups with correlation coefficient 0.1 ≤ are shown here). This suggested that the link between high legume intake and high IDP score is not generalizable, but appears to be produced by the particular dietary pattern of the study population.

In our present study, the association between IDP and cognitive decline was observed for reasoning, and there was no dietary effect on verbal fluency. These findings parallel the inverse association of serum IL-6 with reasoning and lack of association with verbal fluency in our earlier study [5]. We believe these consistencies support our hypothesis that IDP affects cognitive function partly through inflammation. Reasoning is a dimension of fluid intelligence and may be more sensitive to minor decline in neuronal functioning and the age of onset in decline is earlier than crystallized intelligence, such as verbal fluency [30]. In line with this report, the identified association of IDP and reasoning were stronger in the younger age group when it was stratified by median age.

We should consider reverse causation as an alternative explanation for the observed link between dietary pattern and cognitive decline. Such an effect might arise if the presence of sub-syndromal mild cognitive impairment led both to a low-grade inflammatory state and to the dietary pattern we have identified here. Our study participants are relatively healthy and young, and therefore it appears unlikely that preclinical disease might generate the links of the small degrees of cognitive decline we observed with IL-6 and dietary habit. In addition, the association was the clearest in younger participants. Reverse causation, whereby cognitive decline may affect dietary habits, is unlikely to be important in this group, and the dietary effect on cognitive decline probably drives the association. Furthermore, after excluding the participants with the bottom 10% of global cognition score at baseline, results did not change.

The strength of this study includes the large sample size and repeat measurements of dietary intake, IL-6 and cognitive function. Participants were prospectively followed over 10 years with three measurements of cognitive function. IL-6 was measured twice, at a five-year interval. To avoid misclassification and improve validity of dietary intake derived from food frequency questionnaire, we used the average intake from 1991 - 1993 and 1997–1999. Our study also has limitations. First, estimated intake of nutrients derived remains subject to errors of recall of average diet in previous year. The validation of food frequency questionnaire using biomarkers and a 7-day estimated food diary showed a relatively good performance in our cohort [17]. Second, factor loadings are comparatively low for all food groups. This is a feature of the reduced rank regression model, in which the “dietary pattern extracted by reduced rank regression methods explains more variation in response variables [IL6] than any other linear function of predictors but possibly explains only a moderate fraction of predictor variation [food groups]” [18]. However, IDP score tertile identified proportional increases in inflammatory marker levels over the study period (Table 2). Although the factor loading is low, the reduced rank regression model enabled us to identify the existence of inflammatory diet patterns in our participants. Lastly, there were no significant difference in age between excluded and included participants. Excluded participants tended to be female, to have lower education level and occupational status compared to included participants. Cognitive function scores were lower in the excluded participants except memory score which did not differ between the groups. Thus, there is a possibility of some selection bias.

In conclusion, an IDP characterized by high intake of red meat, processed meat, and peas and legumes, and low intake of whole grain, was associated with high circulating IL-6 levels and accelerated cognitive decline over a 10 year follow-up. Further research in other cohorts is needed to replicate our finding, followed ideally by intervention studies to determine whether the avoidance of IDP would reduce inflammation and, in the long term, slow future cognitive decline.

## Funding support

This study was supported in part by UNESCO- L'Oreal Foundation, Astellas Foundation for Research on Metabolic Disorders, the British Medical Research Council (K013351), the Economic and Social Research Council and the National Institute on Aging (R01 AG034454). Archana Singh-Manoux receives research support from the National Institutes of Health (National Institute on Aging R01AG013196 (PI), R01AG034454 (PI)). Eric Brunner is supported by the British Heart Foundation (RG/13/2/30098).

## Authors' contributions to manuscript

Mio Ozawa contributed to the study concept, design, interpretation of data, statistical analysis, and writing the manuscript. Martin Shipley contributed to the study concept, interpretation of data, statistical analysis, and writing the manuscript. Mika Kivimaki, and Archana Singh-Manoux are study coordinators and contributed to the study concept, interpretation of data, and writing the manuscript. Eric Brunner is a study coordinator and contributed to the study concept, interpretation of data, statistical analysis, and writing the manuscript.

## Conflict of interest

The authors report that there are no disclosures relevant to this publication.

## Figures and Tables

**Table 1:** Factor loadings of food groups associated with inflammatory dietary pattern score.

Food group	Factor loadings^a^^,^^b^
	Diet pattern score^c^
Red meat	**0.27**
Poultry	0.15
Processed meats	**0.25**
Organ meats	0.03
Fish	−0.01
Refined grain	0.13
Whole grain	**−0.47**
Eggs	0.18
Butter	0.17
Margarine	−0.003
High-fat dairy	0.10
Low-fat dairy	0.08
Soya products	−0.06
Liqueurs/spirits	0.10
Wine	−0.23
Beer	0.11
Hot drinks	0.14
Fruits	0.08
Fruit juice	−0.22
Leafy vegetables	0.05
Cruciferous vegetables	0.18
Other vegetables	0.12
Tomatoes	0.10
Peas and dried legumes	**0.25**
Soup	0.10
Nuts	−0.18
Potatoes	0.11
Quiche/pie	0.03
Pizza/lasagne	−0.20
Fried food	**0.25**
Snacks	−0.09
Desserts/biscuits	−0.17
Chocolate and sweets	−0.001
Sugar beverages	0.12
Low-energy beverages	0.000
Condiments	0.07
Salad dressing	−0.22

a Factor loadings represent the magnitude and direction of each food group's contribution to inflammatory diet pattern. A positive factor loading indicates an increased intake of the food group. A negative loading indicates less intake of the food group.

b Factor loadings ≥0.25 or ≤−0.25 are shown in bold.

c Patterns were derived with reduced rank regression with a biomarker (serum interleukin-6) in 1991–1993 and 1997–1999 as response variables and 37 food and food groups as independent variables.

**Table 2:** Characteristics of the study population by tertiles of inflammatory dietary pattern score.

	Diet pattern score			P value
	Tertile 1 (<−0.44) n = 1694	Tertile 2 (−0.44, 0.41) n = 1695	Tertile 3 (>0.41) n = 1694	
IL6 (SD) 1991–1993	1.6 (1.5)	1.8 (1.6)	2.1 (1.8)	<0.001
1997–1999	2.1 (1.9)	2.3 (2.0)	2.5 (2.1)	<0.001
Age, y (SD)	54.9 (6.0)	56.1 (6.1)	56.9 (5.9)	<0.001
Female (%)	408 (24.1)	506 (29.9)	544 (32.1)	<0.001
Caucasian (%)	1607 (94.9)	1584 (93.5)	1494 (88.2)	<0.001
Married/cohabiting (%)	1298 (76.6)	1297 (76.5)	1249 (73.7)	0.05
High occupational position (%)	445 (26.3)	359 (21.1)	201 (11.9)	<0.001
University or higher (%)	749 (44.2)	602 (35.5)	384 (22.7)	<0.001
Leisure time physical activity (%)	957 (56.5)	907 (53.5)	851 (50.2)	<0.001
Systolic blood pressure, mmHg (SD)	121.5 (16.1)	122.9 (16.6)	124.5 (16.6)	<0.001
Diastolic blood pressure, mmHg (SD)	76.9 (10.4)	77.1 (10.4)	78.2 (10.7)	<0.001
Hypertension (%)	406 (24.0)	461 (27.2)	568 (33.5)	<0.001
Diabetes (%)	52 (3.4)	63 (3.7)	107 (6.3)	<0.001
Body mass index, kg/m^2^ (SD)	25.3 (3.1)	25.9 (3.8)	26.9 (4.0)	<0.001
Current smoking (%)	85 (5.0)	123 (7.3)	234 (13.8)	<0.001
Energy intake, KJ/d (SD)	9504.4 (2266.0)	9110.7 (2162.5)	8512.5 (2165.9)	<0.001

**Table 3:** Association of inflammatory dietary pattern score at baseline and cognitive decline over the subsequent 10 years.

	Cognitive change over 10 years, coefficient (95% CI)			p for interaction^a^
	Diet pattern score			
	Tertile 1 (low)	Tertile 2 (middle)	Tertile 3 (high)	
**Adjusted for demographics**				
Reasoning	−0.31 (−0.34, −0.28)	−0.35 (−0.38, −0.32)*	−0.37 (−0.40,−0.34)**	0.01
Memory	−0.25 (−0.30, −0.20)	−0.29 (−0.34, −0.24)	−0.30 (−0.35, −0.25)	0.40
Verbal fluency	−0.38 (−0.42, −0.34)	−0.40 (−0.44, −0.37)	−0.40 (−0.43, −0.36)	0.68
Global cognition	−0.31 (−0.34, −0.29)	−0.35 (−0.37, −0.32)*	−0.35 (−0.38, −0.33)*	0.05
**Adjusted for demographics and health related factors**				
Reasoning	−0.31 (−0.34, −0.28)	−0.36 (−0.39, −0.33)*	−0.37 (−0.40, −0.33)*	0.04
Memory	−0.24 (−0.30, −0.19)	−0.28 (−0.33, −0.22)	−0.29 (−0.35, −0.24)	0.45
Verbal fluency	−0.37 (−0.41, −0.33)	−0.40 (−0.44, −0.36)	−0.40 (−0.44, −0.36)	0.44
Global cognition	−0.31 (−0.33, −0.28)	−0.35 (−0.37, −0.32)*	−0.35 (−0.38, −0.32)*	0.04

Abbreviation: CI, confidence interval.

NOTE: Estimates derived from linear mixed models using three assessments over 10 years.

Models adjusted for demographics include age, sex, ethnicity, occupational position, education, and total energy intake at baseline.

Health related factors include body mass index, diabetes mellitus, hypertension, smoking history, and leisure time physical activity at baseline.

**p < 0.01, difference in mean cognitive decline compared with referent tertile 1.

*p < 0.05, difference in mean cognitive decline compared with referent tertile 1.

a The interaction term tested whether cognitive decline differed across the 3 tertiles of diet pattern score (i.e. low, middle, and high).

**Table 4:** Association of inflammatory dietary pattern score at baseline (mean of measures in 1991–1993 and 1997–1999) and decline of 3 points or more in MMSE (2002–2004, 2007–2009).

	Diet pattern score			p for trend
	Tertile 1 (low)	Tertile 2 (middle)	Tertile 3 (high)	
**Adjusted for demographics**				
OR (95% CI)	1.0	1.64 (1.05–2.57)	1.94 (1.24–3.03)	0.004
**Adjusted for demographics and health related factors**				
OR (95% CI)	1.0	1.46 (0.90–2.34)	1.37 (0.85–2.22)	0.25

Abbreviation: OR, odds ratio; CI, confidence interval.

Total n = 3917, decline in MMSE score ≥3 points: n = 170.

Models adjusted for demographics include age, sex, ethnicity, occupational position, education, and total energy intake.

Health related factors include body mass index, diabetes mellitus, hypertension, smoking history, and leisure time physical activity.
