# Supplementary material for: Dietary pattern, inflammation and cognitive decline: The Whitehall II prospective cohort study
Source: Clin Nutr. 2017 Apr;36(2):506–12. doi: 10.1016/j.clnu.2016.01.013 (PMC5381339; doi:10.1016/j.clnu.2016.01.013)
Supplement: Supplementary file 1 [file mmc1.pdf]

Supplemental Table 1. Correlation coefficients between food groups and interleukin-6

Food Group	IL-6 (1991-1993)	IL-6 (1997-1999)
Red meat	0.05	0.05[a]
Poultry	0.03	0.02
Processed meats	0.03	0.05[a]
Organ meats	0.002	0.009
Fish	0.009	-0.0003
Refined grain	0.006	0.003
Whole grain	-0.04	-0.08[a]
Eggs	0.04	0.04
Butter	0.02	0.04
Margarine	-0.01	-0.01
High-fat dairy	0.008	0.03
Low-fat dairy	0.01	0.0005
Soya products	-0.002	-0.007
Liqueurs/spirits	-0.01	0.03
Wine	-0.01	-0.03
Beer	-0.02	0.02
Hot drinks	0.03	0.01
Fruits	0.02	0.01
Fruit juice	-0.03	-0.04
Leafy vegetables	0.005	0.02
Cruciferous vegetables	0.02	0.01
Other vegetables	0.020	0.007
Tomatoes	0.03	0.006
Peas and dried legumes	0.02	0.05[a]
Soup	0.02	0.03
Nuts	-0.03	-0.03
Potatoes	0.02	0.0006
Quiche/pie	-0.02	-0.02
Pizza/lasagne	-0.05[a]	-0.07[a]
Fried food	0.07[a]	0.07[a]
Snacks	-0.002	-0.04
Desserts/biscuits	-0.05	-0.04
Chocolate and sweets	-0.01	-0.02
Sugar beverages	0.03	0.04
Low-energy beverages	.	.
Condiments	0.01	-0.002
Salad dressing	-0.05[a]	-0.07[a]

[a]$p<0.001$

Supplemental table 2. Association of inflammatory diet pattern score at baseline and cognitive decline over the subsequent 10 years stratified by median age(=56).

	Cognitive change over 10 years, coefficient (95% CI) Model 1			p for interaction†	Cognitive change over 10 years, coefficient (95% CI) Model 2			p for interaction†
	T1 (low)	T2 (middle)	T3 (high)		T1 (low)	T2 (middle)	T3 (high)	
**Age<56**								
Reasoning	-0.28 (-0.33, -0.23)	-0.36 (-0.41, -0.30)**	-0.36 (-0.41, -0.30)**	0.005	-0.29 (-0.35, -0.23)	-0.37 (-0.43, -0.31)**	-0.35 (-0.41, -0.29)*	0.009
Memory	-0.28 (-0.38, -0.18)	-0.35 (-0.45, -0.25)	-0.33 (-0.43, -0.23)	0.33	-0.26 (-0.37, -0.16)	-0.33 (-0.43, -0.22)	-0.32 (-0.43, -0.22)	0.36
Verbal fluency	-0.38 (-0.45, -0.31)	-0.40 (-0.47, -0.33)	-0.37 (-0.42, -0.31)	0.69	-0.35 (-0.43, -0.27)	-0.39 (-0.47, -0.31)	-0.35 (-0.43, -0.27)	0.54
Global cognition	-0.31 (-0.35, -0.26)	-0.37 (-0.41, -0.32)**	-0.34 (-0.39, -0.29)	0.02	-0.29 (-0.43, -0.27)	-0.39 (-0.47, -0.31)**	-0.35 (-0.43, -0.27)	0.01
**Age≥56**								
Reasoning	-0.31 (-0.38, -0.25)	-0.30 (-0.37, -0.24)	-0.35 (-0.41, -0.29)	0.29	-0.32 (-0.39, -0.25)	-0.31 (-0.38, -0.13)	-0.35 (-0.42, -0.28)	0.52
Memory	-0.24 (-0.36, -0.13)	-0.26 (-0.37, -0.15)	-0.27 (-0.39, -0.16)	0.85	-0.26 (-0.39, -0.13)	-0.26 (-0.38, -0.14)	-0.27 (-0.39, -0.15)	0.98
Verbal fluency	-0.35 (-0.43, -0.27)	-0.38 (-0.46, -0.30)	-0.41 (-0.48, -0.33)	0.43	-0.33 (-0.42, -0.25)	-0.37 (-0.45, -0.28)	-0.40 (-0.49, -0.32)	0.31
Global cognition	-0.32 (-0.38, -0.27)	-0.30 (-0.36, -0.25)	-0.35 (-0.40, -0.29)	0.29	-0.31 (-0.37, -0.24)	-0.31 (-0.37, -0.25)	-0.34 (-0.40, -0.29)	0.39

Abbreviation: CI, confidence interval

NOTE: Estimates derived from linear mixed models using three assessments over 10 years.

Model 1 is adjusted for demographics include age, sex, ethnicity, occupational position, education, and total energy intake at baseline.

Model 2 is adjusted as in Model 1 + health related factors include body mass index, diabetes mellitus, hypertension, smoking history, and leisure time physical activity at baseline.

**p<0.01, difference in mean cognitive decline compared with referent tertile 1.

*p<0.05, difference in mean cognitive decline compared with referent tertile 1.

†The interaction term tested whether cognitive decline differed across the 3 tertiles of diet pattern score (i.e. low, middle, and high).

Supplemental Table 3. Association of inflammatory dietary pattern score at baseline and cognitive decline over the subsequent 10 years stratified by median body mass index.

	Cognitive change over 10 years, coefficient (95% CI)			p for interaction†	Cognitive change over 10 years, coefficient (95% CI)			p for interaction†
	Model 1				Model 2			
	Tertile 1 (low)	Tertile 2 (middle)	Tertile 3 (high)		Tertile 1 (low)	Tertile 2 (middle)	Tertile 3 (high)	
**BMI≤26**								
Reasoning	-0.32 (-0.35, -0.28)	-0.34 (-0.38, -0.31)	-0.37 (-0.41, -0.33)*	0.11	-0.30 (-0.35, -0.25)	-0.34 (-0.39, -0.28)	-0.35 (-0.40, -0.29)	0.30
Memory	-0.24 (-0.30, -0.17)	-0.32 (-0.38, -0.25)	-0.33 (-0.41, -0.26)	0.11	-0.20 (-0.29, -0.10)	-0.28 (-0.38, -0.19)	-0.30 (-0.40, -0.20)	0.14
Verbal fluency	-0.37 (-0.42, -0.33)	-0.42 (-0.47, -0.38)	-0.38 (-0.44, -0.33)	0.29	-0.36 (-0.43, -0.29)	-0.43 (-0.50, -0.36)	-0.40 (-0.47, -0.33)	0.16
Global cognition	-0.31 (-0.34, -0.28)	-0.36 (-0.39, -0.33)*	-0.36 (-0.39, -0.32)*	0.03	-0.28 (-0.33, -0.24)	-0.35 (-0.39, -0.30)**	-0.35 (-0.39, -0.30)*	0.02
**BMI>26**								
Reasoning	-0.31 (-0.36, -0.26)	-0.37 (-0.42, -0.32)	-0.38 (-0.42, -0.33)	0.13	-0.31 (-0.37, -0.25)	-0.35 (-0.40, -0.29)	-0.38 (-0.43, -0.32)*	0.11
Memory	-0.27 (-0.36, -0.18)	-0.24 (-0.32, -0.16)	-0.27 (-0.35, -0.19)	0.85	-0.27 (-0.38, -0.17)	-0.24 (-0.33, -0.15)	-0.26 (-0.36, -0.17)	0.86
Verbal fluency	-0.40 (-0.47, -0.33)	-0.37 (-0.43, -0.31)	-0.41 (-0.47, -0.36)	0.56	-0.39 (-0.46, -0.31)	-0.36 (-0.43, -0.29)	-0.40 (-0.47, -0.33)	0.62
Global cognition	-0.33 (-0.37, -0.28)	-0.33 (-0.37, -0.29)	-0.35 (-0.47, -0.36)	0.65	-0.32 (-0.37, -0.27)	-0.33 (-0.37, -0.28)	-0.35 (-0.39, -0.30)	0.69

Abbreviation: CI, confidence interval

NOTE: Estimates derived from linear mixed models using three assessments over 10 years.

Model 1 is adjusted for demographics include age, sex, ethnicity, occupational position, education, and total energy intake at baseline.

Model 2 is adjusted as in Model 1 + health related factors include body mass index, diabetes mellitus, hypertension, smoking history, and leisure time physical activity at baseline.

**p<0.01, difference in mean cognitive decline compared with referent tertile 1.

*p<0.05, difference in mean cognitive decline compared with referent tertile 1.

†The interaction term tested whether cognitive decline differed across the 3 tertiles of diet pattern score (i.e. low, middle, and high).

Supplemental Table 4. Dietary characteristics of the study population by tertiles of inflammatory dietary pattern score

	Diet pattern score		
	Tertile 1 (<-0.44) n=1694	Tertile 2 (-0.44, 0.41) n=1695	Tertile 3 (>0.41) n=1694
Red meat	35.1 (27.2)	46.6 (30.1)	60.4 (40.8)
Poultry	45.7 (31.7)	53.4 (33.2)	60.1 (47.4)
Processed meats	13.6 (10.6)	17.2 (12.1)	23.1 (16.5)
Organ meats	1.4 (2.4)	1.5 (2.1)	1.6 (2.6)
Fish	30.9 (24.5)	30.9 (25.1)	29.6 (27.6)
Refined grain	88.3 (56.9)	95.4 (58.3)	111.2 (71.3)
Whole grain	181.6 (83.8)	126.6 (66.1)	79.0 (56.6)
Eggs	12.2 (11.3)	14.7 (12.6)	18.9 (15.6)
Butter	3.8 (6.5)	5.4 (8.2)	7.8 (11.2)
Margarine	13.3 (11.2)	13.2 (10.9)	13.1 (12.2)
High-fat dairy	66.9 (84.8)	84.0 (106.6)	95.6 (124.8)
Low-fat dairy	247.2 (151.9)	264.8 (182.5)	290.4 (226.1)
Soya products	5.7 (27.5)	3.5 (21.4)	2.2 (13.1)
Liqueurs/spirits	9.2 (13.4)	10.5 (16.3)	13.8 (25.0)
Wine	120.5 (149.3)	82.0 (104.1)	56.6 (81.9)
Beer	118.8 (211.2)	138.0 (268.3)	216.4 (414.5)
Hot drinks	545.1 (383.8)	611.0 (393.3)	719.9 (469.5)
Fruits	299.2 (181.4)	326.7 (223.2)	341.9 (265.9)
Fruit juice	150.1 (138.9)	123.1 (115.1)	84.1 (92.2)
Leafy vegetables	17.3 (13.4)	17.1 (14.9)	18.6 (20.1)
Cruciferous vegetables	56.0 (38.9)	67.5 (45.1)	78.4 (60.0)
Other vegetables	70.2 (44.3)	75.5 (44.3)	84.1 (92.2)
Tomatoes	43.9 (31.5)	47.5 (34.2)	53.1 (50.0)
Peas and dried legumes	50.7 (30.3)	61.1 (32.0)	75.8 (48.9)
Soup	35.5 (38.8)	40.1 (42.2)	46.7 (60.9)
Nuts	6.6 (11.0)	4.2 (6.2)	3.1 (4.6)
Potatoes	87.1 (50.8)	97.8 (58.3)	102.1 (62.3)
Quiche/pie	11.5 (11.2)	12.2 (11.6)	12.4 (13.1)
Pizza/lasagne	25.5 (21.0)	20.6 (17.7)	15.1 (16.1)
Fried food	42.7 (31.2)	54.5 (35.4)	70.1 (46.2)
Snacks	8.1 (11.1)	6.6 (8.7)	5.8 (8.1)
Desserts/biscuits	65.2 (47.4)	59.6 (40.6)	46.4 (34.3)
Chocolate and sweets	32.0 (22.4)	34.1 (24.4)	32.0 (24.2)
Sugar beverages	50.6 (84.3)	67.7 (101.3)	82.1 (123.7)
Low-energy beverages	0	0	0
Condiments	20.8 (17.8)	23.4 (20.7)	24.5 (23.2)
Salad dressing	7.7 (7.7)	5.6 (5.9)	3.7 (4.8)
